# Hidden Realities of Infant Feeding: Systematic Review of Qualitative Findings from Parents

**DOI:** 10.3390/bs10050083

**Published:** 2020-04-27

**Authors:** Anne M. Dattilo, Ryan S. Carvalho, Rubens Feferbaum, Stewart Forsyth, Ai Zhao

**Affiliations:** 1 Nestlé Nutrition, Avenue Nestle, 55 CH-1800 Vevey, Switzerland; ryan.carvalho@nestle.com; 2 Children’s Institute University of São Paulo, Rua Tremembé, São Paulo-SP 01256-010, Brazil; rubens.feferbaum@hc.fm.usp.br; 3 School of Medicine, University of Dundee, Dundee DD5 1JG, UK; jsforsyth@dundee.ac.uk; 4 School of Public Health, Peking University Health Science Center, Xueyuan Road No. 38., Beijing 100191, China; aizhao@bjmu.edu.cn

**Keywords:** infant feeding, parents, behavior, qualitative methods, nutrition education, systematic review

## Abstract

A growing, global conversation, regarding realities and challenges that parents experience today is ever-present. To understand recent parent’s attitudes, beliefs, and perceptions regarding infant feeding, we sought to systematically identify and synthesize original qualitative research findings. Following the Enhancing Transparency in Reporting the Synthesis of Qualitative Research (ENTREQ) framework, electronic databases were searched with a priori terms applied to title/abstract fields and limited to studies published in English from 2015 to 2019, inclusive. Study quality assessment was conducted using the Critical Appraisal Skills Programme (CASP) checklist, and thematic analyses performed. Of 73 studies meeting inclusion criteria, four major themes emerged. (1) Breastfeeding is best for an infant; (2) Distinct attitudes, beliefs, and perceptions of mothers that breastfeed, and those that could not or chose not to breastfeed, are evident; (3) Infant feeding behaviors are influenced by the socio-cultural environment of the family, and (4) Parent’s expectations of education and support addressing personal infant feeding choices from health care providers are not always met. This systematic review, guided by constructs within behavioral models and theories, provides updated findings to help inform the development of nutrition education curricula and public policy programs. Results can be applied within scale-up nutrition and behavioral education interventions that support parents during infant feeding.

## 1. Introduction

Nutrition during the first 1000 days, spanning from conception to age 24 months, has critical influence on the immediate and long-term physical and cognitive development of infants. The period from birth through the first 12 months characterizes a unique time when parents or caregivers make essentially all feeding decisions about what and how their infant is offered food [1]. Although the definition of a modern family is changing [2], parents are currently described as the main caregivers of children in the home [3] and infant feeding is a large component of that care that encompasses the social, cultural, and economic structure of a parent’s life [4].

Significant progress with improved infant feeding and nutrition has been realized through nutrition education efforts, yet childhood growth faltering as evident by the number of children at both the lower and upper percentiles of the World Health Organization growth standards remains a significant public health concern across the globe [5]. Breastfeeding rates are below global targets [6], particularly in high-income countries [7], and assessment of parental complementary feeding behaviors has identified room for improvement from all regions studied [8,9,10]. Understanding the current modifiable determinants influencing today’s parents feeding choices and behaviors is essential in providing support and education.

Education strategies likely to benefit parents are guided by a theory of health behavior, and evidence indicates that utilization of behavioral models and theories for nutrition education interventions improves effectiveness [11,12]. Within the often applied Social-cognitive Theory, Theory of Planned Behavior, and Health Belief Model, infant feeding constructs (concepts) include parental feeding attitudes, beliefs, perceptions, social norms, environmental constraints, as well as skills and knowledge [13]. Understanding the underlying psychosocial drivers, or “hidden realities” related to infant feeding behaviors of parents provides insights for developing, improving, and scaling nutrition education interventions. Ethnographic and qualitative research methods are well suited to capture these social-cognitive constructs [14,15].

Meta-synthesis of qualitative studies and systematic qualitative reviews have previously contributed to an understanding of parent’s perspectives on infant and child dietary patterns unique to low- and middle-income countries [16,17,18]. Feeding experiences of migrant and refugee women in Australia have been assessed by qualitative synthesis of publications through 2014 [19], and men’s views, perceptions, and experiences with infant feeding have been recently summarized [20,21]. In addition, via a systematic qualitative review of studies through 2014 [22], knowledge has been expanded related to factors that influence parent’s timing, choices, and process of transitioning their infant’s diet to family foods. Including a majority of studies published through 2015, parent’s experiences and perceptions of complementary food and feeding recommendations have also been reviewed [23]. Meta-ethnographic and systematic qualitative reviews have specifically addressed mother’s experiences with breastfeeding [24,25,26], yet references within these reviews may not reflect current social-cognitive constructs associated with infant feeding of modern parents, as reviews have not included studies published after 2015. As such, an update to previous research syntheses is needed to investigate if there are new developments within more current literature.

The aim of this study was to provide a current and comprehensive synthesis of original qualitative literature findings related to parent attitudes, beliefs, and perceptions regarding infant feeding and to identify factors that influence parent infant feeding decisions. As only studies published between 2015 and 2019 are included, our results provide a new assessment of the most recent parent perspectives of infant feeding.

## 2. Materials and Methods

Guidelines from the Enhancing Transparency in Reporting the Synthesis of Qualitative Research (ENTREQ) statement [27] were followed within this qualitative review synthesis. The Preferred Reporting Items for Systematic reviews and Meta-Analyses (PRISMA) flowchart was utilized for reporting the different phases of searching, screening and identifying studies for inclusion in the qualitative synthesis [28].

### 2.1. Search Strategy and Study Selection

A pilot literature search strategy with terms of “infant, feeding, perception, attitude, and belief” was conducted in March 2019 to provide an initial overview of the literature and to help inform the final search strategy. The search terms and process utilized in the final search strategy included: infant* AND parent* OR mother* OR father* OR caregiver*; AND feeding* OR “feeding behavior*” OR “infant feeding” OR breastfeed* OR breast feed* OR bottlefeed* OR “bottle feed*” OR formula* OR “infant formula” OR “baby formula” OR wean* OR “complementary feeding” OR “baby food*”; AND perception* OR attitude* OR belief* OR perspective* OR view* OR emotion* OR influence* OR feel* OR view*; AND qualitative OR “qualitative study” OR “qualitative analysis” OR “qualitative interview” OR “qualitative research” OR ethnograph* OR “thematic analysis” OR “focus group*” OR interview*. The search strategy was applied to electronic scientific databases of Medline, PsycInfo, and Cochrane Database of Systematic Reviews, with limits on year (2015–2019, inclusive) and English language. Reference lists of recently published studies were hand searched for additional potential inclusions.

Studies were required to have enrolled a parent or primary caregiver of an infant up to 1 year of age, and have a focus on infant feeding. Included studies were required to have utilized qualitative data analyses; if mixed methods were reported in an individual study, findings from qualitative components were included. Any discrepancies with study inclusion were discussed by authors and resolved by consensus. Excluded studies were those that enrolled preterm infants, infants with morbidities, or if the study enrolled only adolescent age mothers, pregnant women, HIV+ mothers, or women with a history of infertility. Studies that addressed baby-led weaning only, or with publication dates < 2018 that were specific to fathers only were excluded, as recent reviews have included such findings.

### 2.2. Study Quality Assessment and Data Reporting

Studies were evaluated for quality and internal validity using the Critical Appraisal Skill Programme (CASP) tool for qualitative research [29]. Completeness of reporting and potential of bias were addressed within the tool, as well as appropriateness of study design, methods, data collection, and analysis methods used. Since CASP does not use assessment scores, we adopted a 3-point rating system similar to others [16,17,19,26,30,31]. For each checklist item, studies were scored with 2 points if a CASP criterion was met, 1 point if unable to determine, and 0 points if the standard was not met. Any disagreements in quality appraisal were resolved by author discussion. As there is no consensus about which, if any, quality criteria should be applied to qualitative research synthesis [31], and due to the risk of losing new insights [22,23], quality was not used as an exclusion criterion in the current review.

Thematic synthesis [32], as utilized in other qualitative reviews [16,17,18,22,23], was the qualitative evidence synthesis method employed. This approach is designed to identify new themes and concepts, while maintaining conclusions of the individual primary study. The process included becoming familiar with the data by open-minded reading of each study, line-by-line extraction and coding of study findings and organization into first-order descriptive themes, sub-theme, and higher order major themes. After coding results of selected studies, any disparities were addressed by author discussion.

## 3. Results

**Figure 1:**
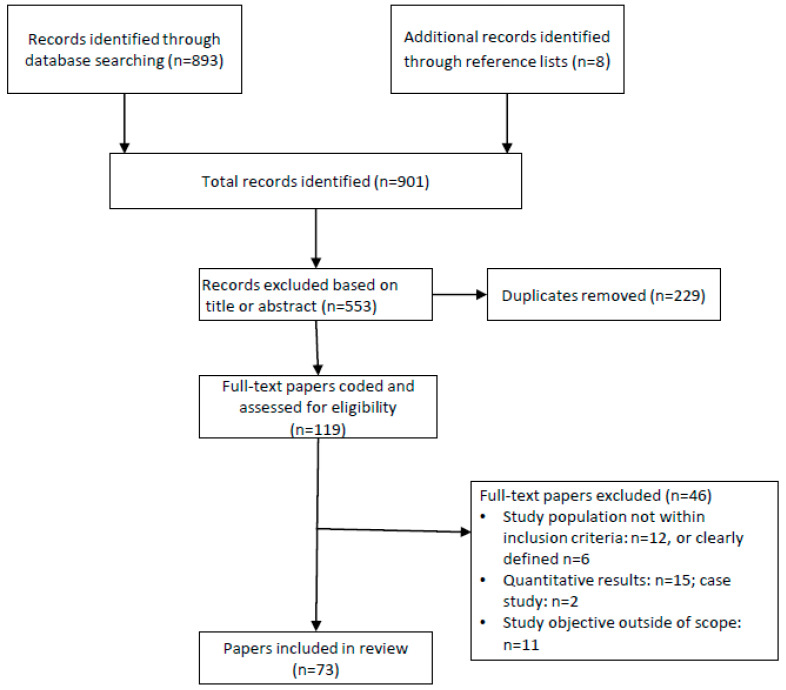
Systematic review flow diagram for study selection.

The literature search identified 901 unique papers published between 2015 and 2019 that potentially brought insight to parent’s attitudes, beliefs, and perceptions and influencers regarding infant feeding behaviors. Following title and abstract screening against inclusion criteria, full texts of 119 papers were coded and assessed for eligibility. After removing 46 studies not meeting inclusion criteria, 73 original qualitative studies served as the base for this review. Details of the studies screened, included, and excluded are on Figure 1.

More than half (55%) of the studies were published in the past 3 years; 2019 (n = 17), 2018 (n = 11), 2017 (n = 12), 2016 (n = 18) to 2015 (n = 15). A majority (82%) of the studies were conducted with parents from North America (n = 28), Europe (n = 10), United Kingdom (10), and Australia (n = 12). A limited number of studies included parents from Asia (n = 8), or Africa (n = 5). Focus groups (n = 28) and interviews (n = 45) were most often utilized as methods of data collection, and some studies included a variety of methods. Studies were dominated by the experiences with milk feeding (n = 55), and the majority enrolled only mothers (n = 52). Details of the 73 studies included in the systematic literature review synthesis are identified in Supplementary Materials.

Study quality assessment ratings were generally moderate-to-high, and all study scores met at least 16 of 20 points. Of the studies with the lowest quality rankings (n = 7), most met at least 8 of the 10 criteria within the CASP [29], with exceptions in categories of incomplete details provided within research description approach, and disclosure of the relationship between researcher and participant. Aside from publication year, no differences were identified within studies of moderate quality (e.g., 16 points) of which were published prior to 2017, compared to those of higher quality (20 points). Ethical standards were addressed in all of the studies, and no significant failings within methods or analyses were detected within any of the included studies.

Thematic analyses identified four major themes. (1) Breastfeeding is best for an infant; (2) Distinct attitudes, beliefs, and perceptions of mothers that breastfeed, and those that could not or chose not to breastfeed, are evident; (3) Infant feeding behaviors are influenced by the socio-cultural environment of the family, and (4) Parent’s expectations of education and support addressing personal infant feeding choices from health care providers are not always met.

### 3.1. Breastfeeding Is Best for an Infant

Parents perceived that “breastfeeding is the best way to feed infants”, despite if they had personal breastfeeding experience or not [33,34,35,36,37,38,39,40,41,42,43,44,45,46,47,48,49,50,51,52,53,54,55,56,57,58]. This finding was consistent for parents from studies within all geographical regions, with the exception of two studies in which some mothers reported that breastfeeding (BF), in general, was not acceptable for infant feeding [59] or that colostrum was not considered appropriate [60]. Results from several studies indicated that parents believed that “breastfeeding is the natural way to feed infants” [34,47,56,61], or “the normal way to feed” [62], and that BF is the healthier option for infant milk feeding [41,55,63].

### 3.2. Distinct Attitudes, Beliefs, and Perceptions of Mothers That Breastfeed, and Those That Could Not or Chose Not to Breastfeed, Are Evident

Positive attitudes, beliefs, and perceptions toward BF were frequently reported [40,57,64], such as “breastfeeding creates happiness” [45], and “breastfeeding was a satisfying experience” [65], yet few studies identified positive descriptors from women that chose not to BF [64]. Overall, studies more often reported negative terminology of constructs as parents described their feeding experiences.

Studies that included mothers that BF reported “negative feelings of judgement from others” [35,54,66,67,68], and several identified “stigma, shame, and personal embarrassment” to feed in public [39,69,70], which may have contributed to their reported sense of isolation [38,67]. Some women described shame as experienced and internalized through exposure of their body [67] or a negative body image [59]. Feelings of guilt for not finding BF easy [38], for taking time out of work to BF [66], or for continuing to BF although finding the practice aversive [71] were reported. Some mothers felt overwhelmed, anxious, and frustrated with the intensity and unpredictability of breastfeeding [37] and found that “breastfeeding was demanding, not as easy as it should seem, and required perseverance” [72]. Developing resilience to judgement, and recognizing that “everyone has something to say about breastfeeding” [35,66,73] are coping skills that mothers reportedly used to help maintain their BF goals.

One study identified mothers of whom intended to not BF due to being fearful of the practice, or a perception that their behaviors were incompatible with BF, and these mothers were comfortable with their choice [64]. However, the majority of studies with mothers that could not, or elected not to BF, particularly for the duration they intended, reported “feelings of shame, guilt, or stigma” [33,34,65,67,73,74,75,76]. The idealism of “striving to be a good mother” via BF [36,42,55,58,67,68,75,77] created conflict, with potential negative influences on a woman’s self-perception of what it means to be a “good mother”. These findings highlight the divide between perceptions of infant feeding idealism and the reality experienced by many parents.

Studies that included mothers that exclusively BF, or BF for longer durations than typical in their culture, had high internal perceptions of their confidence and determination with their BF decision, despite some challenges in reaching their goals [35,64,78]. Mothers described individual (e.g., determination, self-efficacy for BF) and interpersonal (e.g., social support) coping resources as facilitators of BF maintenance [33,34,53,73]. Social support, “particularly enlisting a female relative, friend, or partner was important for BF continuation” [39,53,56,72,79,80], and one study [81] identified social media as a maternally perceived facilitator of BF duration and maternal support.

Other studies described that mothers differed in their BF practices depending on whether their attitudes and beliefs were infant centered (more likely to BF) or maternal centered (less likely to BF) [48,50,54]. Some mothers perceived that their diet may be nutritionally inadequate to support BF [46,54] or believed that “exclusive BF provided insufficient nourishment for their infant” which led to the early introduction of complementary feeding [43,80,82]. Perceived insufficient BM production was most frequently reported as an influencing factor for cessation of BF [40,42,46,48,54,55,56,83,84,85,86,87].

### 3.3. Infant Feeding Behaviors Are Influenced by the Socio-Cultural Environment of the Family

Although a father’s role in parenting may be changing, studies within this review primarily recruited mothers, and identified mothers as the primary managers of infant and young child feeding [80,88]. Fathers deferred infant feeding decisions to the mother, valued BF and believed it as healthy and natural for babies. As some fathers had seen their partners struggle with BF, they acknowledged that BF was more difficult than they had perceived [62] and some viewed BF as a potentially harmful practice for mothers [61]. Studies that included co-parents reported parental agreement that BF affected the relationship with their infant in different ways, and negotiated with adapting and acceptance of different feeding roles [47]. Involvement in feeding over the first few years was described in terms related to “including ongoing discussions and collaborations around co-parenting related to feeding” [88,89].

Studies that included other family members of the mother [55,69,70,80,84,89,90] identified that infant feeding attitudes, beliefs, and perceptions may be “generationally passed down” and potentially impact infant feeding beliefs and behaviors [59]. This finding was evident by the reported influence of family elders and grandmothers [43,87,89]. Overall, the influence of a mother’s immediate family on her infant feeding decisions and behaviors reportedly had a strong impact [49,84,85]. Advice from family was often contradictory to nutrition-based feeding guidelines, and to show respect to family members, some mothers incorporated family advice instead of recommended practices [91].

Family, tradition, and culture (social norms within the parent’s environment) shaped parental infant feeding beliefs and perceptions about when to begin complementary feeding, and what first foods to offer. Of the studies in this review addressing introduction of solid or semi-solid foods, “beliefs, values, and perceived norms” were a central influence on complementary feeding practices [43,44,49,51,55,59,85,88,89,91,92,93,94,95,96,97,98,99], which brought challenges to immigrant mothers of children who were culturally separated [100,101]. Parents perceived that “everyone gives you advice” [102], and complementary feeding was viewed as a natural progression with the goal of enjoyment of food and development of an expansive palate [95]. Considerations of infants’ own preferences [93], as well as responsiveness to family needs and wants [92] were determinants of food choices. “Cost, location, and access to fresh and traditional foods” [85,93,96] was a priority. Some parents reported dissatisfaction with the “one size fits most” approach of infant feeding guidance as “every child is different” [97,102] and reported relying on their own instincts, or cultural familiarity when deciding what and how to feed their infant.

### 3.4. Parent’s Expectations of Education and Support Addressing Personal Infant Feeding Choices from Health Care Providers Are Not always Met

Parents desired professional and individualized instruction regarding infant feeding that was in keeping with their attitudes, beliefs, culture, and feeding decision from various sources [83], including physicians, pediatric nurses, lactation consultants, or professionals working in health care centers or public nutrition programs, described collectively here as health care providers (HCP). In contrast, studies illuminated that many parents found “infant feeding advice, support, and education from their HCP inadequate, missing completely, inconsistent or contradictory” [36,37,38,41,42,44,46,49,50,51,53,55,57,63,72,74,76,83,97,98,103,104]. As identified within some studies, while it is important to promote and maintain BF, it is also necessary to ensure that the care, education, and needs of parents and their infants that are not BF are met [74,76], without stigmatizing parents who do not BF [68]. Some parents expressed distrust of the feeding information and recommendations provided by HCP and looked to family or peers for more culturally sensitive and practical infant feeding advice [41,100].

A need for strategies and support that “address parent’s personal, cultural, and ideological constraints with infant feeding” were identified within several studies [33,51,56,67,74]. Additionally, a desire for expanded infant nutrition education that included parent’s wider community such as family members, rather than only mothers, was identified within some studies [67,79,80,105]. Role models and support groups were noted as important by parents, but perceived as inadequate [38,72,103].

## 4. Discussion

At the individual or parent level, nutrition education focuses on building a person’s capacities for adoption or change of nutrition-related behaviors conducive to health and wellness. Previous research has suggested that infant feeding is likely to be predicted by socio-cognitive variables [11,12,13], and within this qualitative review we examined mediators and cognitive constructs that potentially influence parent’s infant feeding behavior by identifying their infant feeding attitudes, beliefs, and perceptions. Findings are directly applicable within a nutrition education theoretical framework aimed at improving parental infant feeding behaviors for better health and nutrition of infants and young children.

Results from this review identified that parents predominately agree that breastfeeding is the best way to feed infants. As similar to conclusions from older systematic reviews [24,25], recent mothers described breastfeeding in terms of their “perceived expectations, compared to the reality they experienced.” Similarly, a dichotomous desire to be a good/perfect mother (compared to feeding approaches perceived inconsistent with “good mothering”) [22,25] was realized in the current review.

Although some large studies have reported that mothers often decide about infant feeding on their own initiative [106], previous qualitative reviews have concluded that family and cultural practices are strong influences on infant feeding behaviors [16,17,18,19,23,26]. Our results expand upon previous themes with specific new findings. In particular, parents report a desire, and have expectations, that they will be offered factual education related to their individual and personal infant feeding choices, provided with sensitivity, in a non-judgmental manner. Education and support that addresses family and cultural priorities that empower parents to adopt recommended infant feeding guidance, while preventing or addressing internalized feelings of shame or guilt provides an unmet opportunity within nutrition education.

The current qualitative review was performed according to accepted guidelines [27], appropriate thematic synthesis methods [32], and detailed inclusion of individual study objectives, methods, and results provided (Supplementary File 1). In addition, following the CASP tool for qualitative research [29], individual study quality was rated as moderate-to-high, increasing our confidence within the inputs to this synthesis. Moreover, this review included only studies published within the last five years. As such, the findings of this review represent a methodologically sound and comprehensive synthesis of the most recent parent perspectives regarding “hidden realities” with infant feeding that can be incorporated within behavioral based nutrition education efforts.

Given the current light that this literature on parent’s attitudes, beliefs, and perceptions of infant feeding contributes, this thematic synthesis is not without its limitations. Firstly, the majority of studies included in the current review were conducted in the developed world and published in English. Despite this limitation, our results identified that infant feeding behaviors occur via the socio-cultural environment of the family. Given the consistency of this finding, we anticipate that results would not be different if additional studies from more diverse populations were included. Secondly, as studies did not consistently offer author-generated quotations, and there is lack of consensus for identifying the priority of quotes from participants within individual studies, we chose to adapt author conclusions as quotations within this work. Our approach was diligent and consistent with standard qualitative evidence synthesis methods, yet it is possible that some lower order themes were not included. Thirdly, in the majority of studies, the term parent was frequently synonymous with maternal; further research could explore infant feeding constructs with more clearly defined primary caregivers and support persons within the individual qualitative studies. Lastly, most studies addressed perceptions of women that had or were currently BF, directly from their breasts. Few studies addressed participant weight status or other known confounders related to BF. Studies with parents that chose to provide breastmilk by cup or bottle, provide infant formula, or used mixed-feeding methods would provide additional insight.

## 5. Conclusions

Parental infant feeding attitudes, beliefs, and perceptions are influenced at multiple levels, including individual (self-efficacy, determination to meet goals, wanting to be a “good mom”), and external influences (social support, the “village always has an opinion”, family and culture), as well as reported difficulty of finding education resources to overcome challenges. Parents desire factual education and support that addresses their personal feeding choices and ideology, within a culturally sensitive approach from health care providers.

## Supplementary Materials

The following are available online at https://www.mdpi.com/2076-328X/10/5/83/s1, Supplementary File 1: Details of the 73 studies included in the systematic literature review synthesis.
