# Supplementary material for: Hidden Realities of Infant Feeding: Systematic Review of Qualitative Findings from Parents
Source: Behav Sci (Basel). 2020 Apr 27;10(5):83. doi: 10.3390/bs10050083 (PMC7287829; doi:10.3390/bs10050083)
Supplement: Supplementary file 1 [file behavsci-10-00083-s001.pdf]

Supplementary File 1: Details of the 73 studies included in the systematic literature review synthesis

Author Year Country	Study objective	Population	Qualitative Method	Results/Themes	CASP Score †
Appleton J, et al., 2018 [74] Australia	Explore parents' infant feeding practices to understand how parents use infant formula and what factors may influence this practice.	24 mothers	Semi-structured telephone interviews Analyzed using a pragmatic inductive approach to thematic analysis	Themes: Choice of formula – what is on (and in) the tin; Bottle preparation – mostly by the tin; How much and how often; Informal advice; Formal advice; Bottle stigma (where breastfeeding (bf) promoted).<br>Future health care provider (hcp) information on formula should not undermine bf promotion. Additional support for parents' feeding their infants with formula is necessary.	20
Asiodu IV, et al., 2017 [33] USA (California)	Describe infant feeding perceptions and experiences of African American (AA) mothers and their support persons.	14 pregnant women and 8 support persons	Field observations, demographic questionnaires and multiple in-person interviews	Key themes: Best for baby; Normalization and role models; Social support; Fluid social dynamics and resiliency; Seeking support and empowerment; Combination feeding; Stress, shame and guilt.<br>Reasons few mothers exclusively breastfeed (EBF): Life experiences and stressors, lack of bf role models, limited experiences with bf and lactation, and change to the family dynamic. Many expressed guilt and shame for not meeting bf goal. Future interventions to include social media interventions, messaging around combination feeding, and increased education for identified social support persons. More culturally sensitive messaging around bf and lactation.	20
Ayton JE, et al., 2019 [34] Australia (Tasmania)	Investigate mothers' infant feeding experiences (bf/formula milk feeding) with the aim of understanding how women experience cessation of EBF.	127 mothers (half low socio-economic status)	22 focus groups or 19 semi-structured interviews; data collected 2011-2013	Key themes: valuing bf; endurance, grief. Mothers view bf as 'natural' and 'best' and formula milk as 'wrong' and 'unnatural'. Mothers will endure multiple issues (e.g., pain, low milk supply, mastitis, and public shaming) and make use of various forms of social and physical capital to continue to bf. Cessation of EBF results in lingering feelings of grief and failure making it harmful to women's emotional well-being.	20
Barbosa CE, et al., 2017 [64] USA (Virginia)	Identify and understand factors distinguishing low-income AA women who bf the longest (positive deviants) from those who bf for a shorter duration or do not bf.	25 low-income AA women, grouped by infant feeding practice	7 mini-focus groups	Positive deviant women had more schooling, higher income, bf intention, positive bf and unfavorable formula feeding (ff) attitudes, higher self-efficacy, positive hospital and clinic experiences, more EBF, and greater comfort bf in public.<br>Short-term bf women had varied in bf intention and self-efficacy, seemed to receive insufficient professional bf support, and supplemented bf with ff.<br>ff women intended to ff, feared bf, thought their behaviors were incompatible with bf, were comfortable with and found formula convenient, and received strong support to ff.	19
Beck AL, et al., 2018 [92] USA (California)	Elucidate beliefs and practices related to the introduction of solids and solid food feeding in the first year of life.	26 Low-income, Latino parents	Semi-structured interviews	Themes: First foods; Infant feeding strategies; Introduction of high calorie low nutrient foods; Concerns about weight/growth.<br>Reliance on traditional practices in choosing first foods; strong preference for homemade food (often chicken soup + vegs). In general, responsive feeding practices with minority using pressuring. Few used repeated introduction (intro) of unfamiliar foods. High-calorie, low-nutrient foods introduced at 12 months.	20

Begley A, et al., 2019 [102] Australia	Explore mothers' experiences with the introduction of solid food to their child.	42 mothers of children 4-18 months of age	7 focus groups	Themes: Every child is different (judging signs of readiness); Everyone gives you advice (juggling conflicting advice); Go with your gut (being a 'good' mother); It's not a sin to start them too early or too late (guidelines are advice and not requirements).	20
Boak R, et al., 2016 [93] Australia	Describe the experiences of mothers making food choices for their infants.	32 mothers of infants 4-15 months from a range of socioeconomic backgrounds	Semi-structured interviews	Beliefs, values, norms and knowledge - central influence on choices; cost, quality and availability of various food also key factors; influences of time, parents' capacities, social connections and different information sources evident; infants' own preferences and how parents helped infants with learning to eat also key influences on food choices.	18
Brown A, 2016 [105] UK	Examine what new mothers' feel they need in relation to bf promotion and education, strategies they believe worked, and practices they felt restrictive.	1130 mothers with a 0-2 year old, who planned to bf at birth	Questionnaires (with both closed and open-ended questions)	Themes: a move away from the perception that bf is best (rather than normal); emphasis on wider values other than the health benefits of bf; message that every feed, rather than just 6 months of EBF, matters. Mothers highlighted the need for promotion and education to target family members and wider society.	20
Bueno-Gutierrez D, and Chantry C, 2015 [69] Mexico	Identify the main social obstacles to breastfeeding in a low-income population in Tijuana, Mexico.	129 mothers, fathers, grandparents and key informants	6 focus groups (n=53) and interviews (51 mothers, fathers and grandparents, and 25 key informants)	Themes: embarrassment to feed in public; migrant experience; women's role in society; association of formula with higher social status; marketing by the infant food industry; perception of a non-bf culture; and lack of bf social programs.	18
Burns E, and Triandafilidis E, 2019 [66] Australia	Explore Australian women's experiences of bf at one multi-campus university.	79 staff and students in online survey; 10 current or recently stopped bf staff and students	Mixed-methods design involving an online survey and in depth interviews	Themes: University as positive; Finding private and safe spaces for bf; Feeling self-conscious and unprofessional, such as feeling guilty for taking time out to bf. Developing resilience to judgement, that bf on campus requires a "thick skin" and the capacity to not be offended easily.	20
Charlick SJ, et al., 2019 [35] Australia	Present an in-depth, idiographic interpretation of first-time mothers' experience of EBF for six months.	5 new mothers	Face-to-face semi-structured interviews	Themes: EBF is a personal choice (breast milk is best, central to being a mother, personal determination); EBF is harder than expected (demanding); everyone has something to say about bf (judgment).	20
Cheney AM, et al., 2019 [91] USA	Examine the sociocultural factors underlying infant feeding practices.	19 Latina mothers of children 0-2 enrolled in Early Head Start programs over a 1-year period	Focus groups	Themes: Macrosystem and microsystem intersections; Family social and emotional context.<br>Mothers considered both science and family-based recommended feeding practices. Advice from family often inconsistent with science and nutrition-based recommendations (recco's). Some mothers follow family recco's to preserve relationships, while other employ strategies to not offend.	20
Claesson IM, et al., 2018 [36] Sweden	Identify and describe women's experiences with bf.	11 women with obesity that have bf experience	Semi-structured face-to-face interviews Data collected 2-18 months after childbirth	Themes: Bf - a part of motherhood (hoping to bf; best for baby; promotes attachment). The challenges of bf (technique, exposure of body, unmet expectations); Support for bf (lack of support with latching and infant satisfaction; and for nipple soreness, tiredness and unmet expectations for the mom).	20
Coomson JB, and Aryeetey R, 2018 [70]	Assess perceptions and practice of bf in public as well as the drivers of bf in public.	Lactating women (survey n=300; FGD (n=50)	Survey<br>Focus Groups<br>In-depth interviews	Uncomfortable (52%), embarrassed (27%), or stigmatized (15%)<br>Women in FGDs and IDI mentioned that they bottle feed their children in public places as a way to overcome the challenges of bf in public. Women were more	19

Ghana (Accra)		IDI (adult males n=5 and female health workers n=4)		likely to bf in public if they received support from a spouse or other family member.	
Cortés-Rúa L, and Diaz-Gravalos GJ, 2019 [65] Spain	Discover personal experience and feelings of primiparous women who stopped bf before the expected time, and the role of health professionals.	15 mothers	Semi-structured interview	Bf was considered a satisfying experience; it was also sacrificial and painful. Lack of knowledge, insecurity about nourishing infant and physical pain most important reasons for stopping BF. Duality of feelings between the peace of mind afforded by artificial feeding, knowing that their child is well nourished, and the feeling of guilt for not having maintained bf. Mothers perceived that HCP not fully promoting of bf, referring to practices contrary to its establishment and maintenance.	20
Demirci J, et al., 2018 [37] USA	Describe the early bf experience of primiparous women.	35 healthy primiparous women	Mobile app – women texted bf thoughts and experiences through 8 weeks postpartum	Overarching theme: seeking sustainability and validation.<br>Subthemes: "The breaking point, Lessening the burden, Deference to external authority, Blame and absolution, and Tentative confidence."<br>Mothers felt overwhelmed, anxious and frustrated with the intensity and unpredictability of bf and inconsistent professional bf support.	18
DeVane-Johnson S, et al., 2018 [59] USA	Describe cultural factors influencing AA mothers' perceptions about infant feeding.	AA mothers	6 Focus groups	Themes: Historical trauma; Negative body image; Breastfeeding as "nasty". Events, experiences and other phenomena have been culturally, socially and generationally passed down and integrated into families, potentially influencing bf beliefs and behaviors.	18
Fahlquist JN, 2016 [75] Sweden, UK & Netherlands	Achieve qualitative knowledge about the emotions of non-bf mothers	47 mothers	Anonymous web-based qualitative survey	Themes: depression, anxiety and pain, feeling failed as a mother and woman, loss of freedom/feeling trapped, relief and guilt.	18
Fox R, et al., 2015 [38] UK	Examine experiences of bf and bf support among Baby Cafe service users.	51 mothers (36 interviews and 5 focus groups)	In-depth interviews and focus groups (with users of 8 Baby Cafe bf support groups)	Themes: Many women felt that they had been provided unrealistic expectations of bf by HCPs keen to promote the benefits. This left them unprepared when they had pain, problems and restlessness, leading to feelings of guilt and inadequacy over their feeding decisions. Feelings of pressure, guilt, and blame; Conflicting advice and varying levels of support from health professionals, friends and family. Emphasized the importance of social support from other mothers and peer supporters as 'bf models'.	18
Gallegos D, et al., 2015 [39] Australia	Explore the experience of bf among refugee women living in Australia.	31 women with children aged 2 months to 28 yrs, who had been bf	Individual interviews and facilitated group discussions	Four main themes: cultural bf beliefs and practices; stigma and shame around bf in public; ambivalence towards bf and bf support; support of family. Major source of support was women's mothers and aunties; their presence missed with relocation.	18
Groleau D, et al., 2017 [73] Canada	Compare the experience of bf and bf promotion and support services between mothers receiving care from institutions with low and high levels of Baby-Friendly Initiative (BFI) implementation.	52 bf mothers	11 focus groups (with mothers from 6 institutions; 3 with high and 3 with low levels of BFI implementation)	The flexible approach to bf used by BFI services helped to avoid maternal guilt and shame; the shift to focusing on potential barriers and strategies for overcoming them empowered women to negotiate changes in infant feeding with others and self by addressing the embodied experience of a practice that may not feel natural at the beginning. The importance and influence of support (social, family, partner support) in overcoming bf barriers.	20
Gross TT, et al., 2017 [72]	Explore the long-term bf experiences of low-income AA	11 AA women age 18 or older, WIC	Semi-structured in-depth interviews	Themes: deciding to bf; initiating bf; bf long-term; expanding bf support.	20

USA (Georgia)	women using the positive deviant approach.	participant, having bf one child		Influence of social support. Increase the number of AA WIC PCs and lactation consultant to 'role model' bf. More bf support groups for AA women. Bf campaigns using prominent AA celebrities.	
Hansen E, et al., 2018 [61] Australia (Tasmania)	Investigate how fathers view bf.	26 fathers with children < 24 months of age	Questionnaire and either semi-structured one-on-one or group interviews	Themes: Bf as healthy and natural; the value of bf and breastmilk; a pragmatic approach to bf; bf as something achieved or imposed.<br>Fathers valued bf, saw it as healthy and natural for babies and partner's struggle with bf; formula as option when bf not working. Bf problems affect families, not just mothers and infants.	20
Hawley NL, et al., 2015 [40] American Samoa	Examine mothers' feeding experiences, attitudes and beliefs about infant feeding; identify potential barriers to exclusive bf (EBF).	12 mothers at 16-32 days postpartum	18 Semi-structured interviews	Themes: attitudes and beliefs about infant feeding; bf problems; convenience; family involvement. The benefits of bf were well recognized (best way to feed), but the importance of exclusivity was missed. Formula-use was not preferred but considered an innocuous ''back-up option'' where bf was not possible or not sufficient for infant satiety. Identified barriers to EBF included: the convenience of formula; perceptions among mothers that they were not producing enough breast milk; and pain while bf. Influence of parent's mother or sister as important infant caregivers and source of feeding advice.	19
Hounsome L, and Dowling S, 2018 [62] UK	Investigate father's perceptions of their influence on the decision to bf or ff.	6 men	Semi-structured interviews	Themes: deferring of responsibility to the mother (as perceived that the feeding decision power should come from mother); bf as normal practice; change in attitude; involvement in parenting; advantages for the father.	20
Hvatum I, and Glavin K, 2017 [76] Norway	Describe women's experiences with not bf in a bf culture.	12 mothers	Individual, semi-structured interviews	Themes: Desire to adapt to Norwegian culture; feeling as though one was breaking the law; lack of and unbalanced information.<br>Future: HCPs need to provide balanced information on bf and ff.	20
Jessri M, et al., 2015 [100] Canada	Examine Iranian and Arab refugee/immigrant mothers' barriers, experiences, perceptions and cultural norms that influence complementary feeding practices following settlement in Canada from an emic perspective.	Middle Eastern mothers (n = 22) of infants aged less than 1 year	Focus groups	Three layers of influence emerged, which described mothers' process of infant feeding: socio-cultural, health care system and personal factors. Culture was an umbrella theme influencing all aspects of infant feeding decisions. Mothers cited contrary advice, health care professionals' lack of cultural and religious considerations and lack of relevance and practicality of infant feeding guidelines as the main reasons for ignoring infant feeding recommendations.	20
Johnson AM, et al., 2016 [41] USA (Detroit MI)	Explore AA women's bf thoughts, attitudes, and experiences with HCPs and subsequent influences on their bf interest and behavior.	38 pregnant or lactating AA women; racially diverse HCPs	6 membership specific focus groups	Participants generally agreed that bf is the healthier feeding method but perceived that HCPs were not always supportive and sometimes discourage bf. Non-bf mothers often expressed distrust of the info and recco's given by HCPs and relied more on peers and relatives. Perception that HCPs lacked info and skills to successfully engage AA women around bf. Culturally relevant support at multiple levels needed.	20
Johnson KV, et al., 2019 [94] USA	Explore the influences on complementary feeding among Marshallese mothers and caregivers.	27 Marshallese mothers and caregivers	4 Focus groups	Themes: Marshallese bf customs, introduction of solid foods, Marshallese family feeding customs.	19

Kronborg H, et al., 2015 [42] Denmark	Explore mothers' early bf experiences.	108 first-time mothers six months after giving birth	Open-ended question	Dominant themes: (1) On shaky ground, characterized by bf interwoven with mothering, painful bf, and conflicting advice. (2) Searching for a foothold, characterized by reading the baby's cues, concerns about milk production, for or against bf, and looking for professional support. (3) At ease with choice of feeding, characterized by a thriving baby, trust in breastfeeding capability, and approval of feeding preference by hcp. Together these themes and subthemes constituted the overall theme: being on a bf–bonding trajectory.	20
Kuswara K, et al., 2016 [43] Australia	Explore Chinese immigrant mother's experiences of feeding their infant to gain an insight into the factors shaping their decisions and perceptions of infant growth.	36 Chinese immigrant mothers with children aged 0-12 months, living in Melbourne, Australia	Semi-structured interviews	Themes: bf as best and natural, infant formula, introducing solids, perception of infant growth and health, family had strong influence on infant feeding method, negotiating infant feeding choices, hcp support, conflicting information social support and online resources. Influence of grandparents and husbands.<br>For many new mothers, anxiety that EBF provided insufficient nourishment led to the introduction of formula before six months of age.	19
Lindsay AC, et al., 2017 [44] USA (Greater Boston area)	Explore beliefs and infant feeding practices of Brazilian immigrants.	29 Brazilian mothers living in the US for at least 12 months	5 Focus groups + survey	Themes: Bf and complementary feeding beliefs and practices; Family and cultural influences and experiences on bf and complementary feeding (after relocation to different culture); Barriers to bf in the US; Health programs and health care professionals as sources of advice and support.<br>Majority of mothers initiated bf soon after birth, most did not EBF, using mixed feeding approaches; bf along with formula milk; Family and culture influenced mothers' infant feeding beliefs and practices in early intro of solid foods.	20
Leurer DM, et al., 2019 [82] Africa (Tanzania)	Explore mothers infant feeding practices, beliefs, knowledge, and recommendations to support bf.	30 mothers	Interviews	Lactation is universal in this culture; skills and knowledge are passed inter-generationally from the elder women. Liquid and semi-solid supplements provided <6 months; Mothers perceived their milk alone was nutritionally insufficient with maternal dietary deficiencies cited as a factor.	18
Lutenbacher M, et al., 2016 [103] USA	Identify factors that influence and challenge Black women who choose to bf, and supportive strategies that facilitate successful bf experiences.	16 self-identified Black women who were either pregnant or had given birth within the prior 5 yrs	Focus groups	Categories that emerged: Balancing the influences (people, myths and technology); being in the know; critical periods; supportive transitions.<br>Most women experienced little help with bf from hcp or systems.<br>Role models and personalized support were noted as important but lacking. Role of social support, role models, friends and family.	18
Majee W, et al., 2017 [89] USA	Explore potential influences on how co-parents make infant and toddler feeding decisions.	24 mother-father dyads with a 6-36 month old (from two Midwest, rural, university-system pediatric clinics)	Semi-structured interviews	Themes: Interpersonal factors (peer behavior reinforcement, intergenerational gap, dyad and important others infant feeding conflict, conflict resolution pro-activeness); Organizational factors (healthcare provider infant-feeding support, workplace flexibility): and Community factors (public perceptions on bf and social media influence).	20
Maslin K, et al., 2015 [95] UK (England)	Gain insight into parental perceptions of complementary feeding, specifically opinions on commercially produced baby food.	Mothers (n = 24) of infants aged 4–7 months	Focus groups	Three distinct groups of mothers exist; "relaxed", "concerned" and "balanced", which may be influenced by parity, socioeconomic status and previous experience of weaning.<br>Complementary feeding was viewed as a natural process with the goal of food enjoyment and development of a broad palate. Opinions on readymade baby food were influenced by parity, education level and previous experience of weaning.	20

McNamara K, and Wood E, 2019 [99] Central Asia (Tajikistan)	Explore the social dimensions of food taboos and health beliefs and their potential impact on household-level nutrition.	5-12 participants for each of the 13 focus group	Focus group discussions; 7 with in-married women. 4 with mothers-in-law and 2 with men	Themes: food taboos during pregnancy; health beliefs around breastfeeding; food taboos during infancy and childhood; food taboos unspecific to gender or stage in life.	18
Mgongo M, et al., 2019 [45] E Africa (Tanzania)	Explore social and cultural factors that might influence the practice of bf and EBF in Kilimanjaro region.	78 mothers of infants through 12 months.	Focus group discussions; 9 focus groups, with 3 in each district	Feeding practices: Subthemes: bf creates happiness, gift from mother to child; bf can affect mother's appearance; and bf is tiring.<br>Facilitators and barriers: Subthemes: EBF is good for family economy and prevents child sickness, Breastmilk is the only food for infants. Barriers of chango or makekuu (abdominal pain that occurs during or after bf), fear of the evil eye, and burping causing pain to the breast.	19
Mgongo M, et al., 2018 [46] E Africa (Tanzania)	Explored the knowledge, attitude and practices of EBF among mothers.	78 mothers with 0-12 month old infant	Focus group discussions	Themes: bf knowledge, perceived benefits of EBF, reasons for not practicing EBF, multiple sources for bf info, ways to improve EBF practice in the community. Reasons for not bf included poor maternal nutrition, pressure to return to work; lack of support from partner, family members; inadequate knowledge about expressing breast milk and perceived insufficiency of milk supply. Mothers received conflicting advice from a range of sources.	20
Obeng CS, et al., 2015 [63] USA (Midwest)	Explore and further understand how AA women experience bf as well as which constructs could initiate and promote early bf.	20 First-time mothers or mothers with children	Focus groups	Participants felt that there were health benefits to bf, and organizations such as Women, Infants, and Children (WIC) provided support, resources, and practical lactation consultant education. Lack of information, negative perceptions, and unforeseen circumstances were barriers to bf.	16
Omer-Salim A, et al., 2015 [79] India	Explore factors involved in combining bf and employment in the context of six months of maternity leave.	20 first-time mothers with 8-12 month old infant, who had returned to work after six months' maternity leave	Interviews followed a pre-tested guide with a vignette, one key question and six thematic areas	Women managed competing interests to ensure trusted care and nutrition at home; facing workplace conditions; meeting family roles and responsibilities. To navigate these tensions, women used various approaches. Several individual, familial and workplace factors interacted to both hinder and facilitate the process of combining bf and employment. Tension, negotiation and compromise were inherent to the process. Need for information and support for working mothers to address factors at individual, family, and workplace levels to promote bf, in combination with paid maternity leave. Extended family important influencers on maintain bf and employment.	18
Ouyang Y, et al., 2016 [83] China	Explore difficulties and desires of Chinese bf women (Wuhan) and to propose strategies for increasing EBF.	375 primiparous Chinese women – of which 76 were interviewed	Face-to-face semi-structured, in-depth interviews	Women cited a desire for professional and individualized instruction from various sources; reported bf more difficult than expected.	16
Palmqvist H, et al., 2015 [47] Sweden	Describe partners' knowledge and feelings regarding bf and how they reasoned about equality and involvement during the lactation period.	7 male and 2 female partners	Semi-structured interviews	Wish for the child to be bf/get breast milk described the informants' desire that their child was bf and this desire was based on knowledge of benefits of bf but also on intuitive feelings of bf as something natural. The main theme of Effect of bf on fathers/co-mothers described how bf affected the informants and their relationship to the child and the mother in different ways. The main theme Adaptation and acceptance described how informants accepted the impact that bf had and/or adapted to it and continued to wish for the child to receive breast milk.	18

Peacock-Chambers E, et al., 2017 [48] USA	Examine factors that influence maternal infant feeding practices decisions over time in a diverse inner-city population.	20 mothers (recruited from 2 urban primary care clinics)	Open-ended interviews and demographic questionnaires	All women initiated bf and 65% reported perceived insufficient milk (PIM). An association between PIM and behavioral control emerged as the overarching theme impacting early bf cessation and evolved over time	20
Pineros-Leano M, et al., 2019 [96] USA	Understand how first-generation Latina mothers make feeding decisions for their children.	29 immigrant mothers from Latin American countries (19 from Mexico, 4 from Central America and 6 from South America) with preschoolers enrolled in WIC	Semi-structured interviews	Themes: culture as all-encompassing; location and access to fresh and traditional foods; disjunction between health provider advice and cultural knowledge; responsiveness to family needs and wants as determinants of food choices; intrapersonal conflict stemming from childhood poverty and food insufficiency Latina immigrant mothers engaged in a difficult and even conflicting process when deciding how to feed their children.	19
Radwan H, and Sapsford R, 2016 [49] United Arab Emirates	Identify and explore the perceptions and views that influence mothers' infant feeding and weaning decision.	45 mothers with infants 6 months-2 years	In-depth interviews	Themes: influences of others on the decisions to bf; sources of information (some non-satisfaction with info in maternity ward or about bf complications); infants' behavior; participants' views and decisions about when to introduce supplementary feeding; knowledge of and attitudes toward current WHO recco's; mothers' perception of the benefits of bf; bf tradition of study population.	17
Radzyminski S, and Callister LC, 2016 [50] USA (District of Columbia)	Investigate beliefs, attitudes, and decisions of both bf and ff mothers to document maternal perceptions of the infant feeding decision-making process.	152 postpartum women who had given birth within the past 72 hrs	Interviews	Categories identified as influencing maternal decision making: considering infant nutritional benefits; considering maternal benefits; gaining knowledge about infant feeding; describing personal and professional support.<br>Mothers differed in their choice depending on whether they were infant- or maternal-centered. Most combine bf and ff.	16
Rahman A and Akter F, 2019 [84] Bangladesh	Explore reasons behind infant ff practices from mothers, caregivers, and health care provider's perspective.	Mothers, fathers, Caregiver (grandmother, aunty, sister and cousin sister), HCP	Focus Group: FGD (12)<br>In-dept interviews: IDI (4)<br>Key information interview: KII (12)	Women could not differentiate between formula and other milk.<br>Other reasons for using formula: poor bf practices, lack of appropriate bf practices, superficial knowledge on harmful effect on infant formula, perceived insufficient breast milk production, the influence of family and society, and authoritarian power of hospital staff.	17
Ranch MM, et al., 2019 [104] Sweden	Explore first-time mothers' experiences of the bf support offered by pediatric nurses, as well as to develop and evaluate the Mother Perceived Support from Professionals (MoPPS) scale.	9 first-time mothers with infant 3-5 months of age	Semi-structured interviews	When wanting to bf, mothers had a desire to be offered professional bf support. Perceived support from pediatric nurses was often poor, unhelpful with contradictory advice. Not trusting hcp advice, not encouraged to ask about bf, solving bf problems by themselves from lack of support. In contrast, results showed that mothers felt they could ask for help, when perceived that nurses had good knowledge regarding bf.	17
Russell CG, et al., 2016 [51] Australia	Explore the precursors of key obesity promoting infant feeding practices in mothers with low educational attainment.	29 mothers with infant 2-11 months of age	One-on-one semi-structured telephone interviews	Main themes: a) initiating bf/ff; b) prolonging bf/replacing breast milk with ff; c) best practice formula preparation/sub-optimal formula preparation; d) delaying the introduction of solids foods until around six months of age/introducing solids earlier than four months of age; e) introducing healthy first foods/introducing unhealthy first foods; f) feeding to appetite/use of non-nutritive feeding.	19

Savage JS, et al., 2016 [97] USA (Pennsylvania)	Describe themes characterizing feeding behaviors of women participating in the Special Supplemental Nutrition Program for Women, Infants, and Children (WIC), and identify attitudes, beliefs, and sources of information that inform these practices.	68 low-income women 18 years of age or older, with a child less than 2 years old, and who introduced formula to their child before 1 month	Focus groups and semi-structured individual phone interviews	Themes identified: mothers reported receiving conflicting messaging/advice from medical professionals, WIC nutritionists, and family members, which was confusing; reported dissatisfaction with the "one size fits most" approach; reported relying on their "instincts" and that "all babies are different" when deciding and rationalizing what feeding guidance to follow.<br>Mothers reported feeding behaviors inconsistent with guidance from WIC and pediatric society recco's.	18
Schafer EJ, et al., 2019 [52] USA (Iowa)	Understand gap between bf initiation and duration for first-time mothers.	28 first-time mothers	Semi-structured interviews	7 turning points identified bf problems, the unknowns, recurring stressors, living new life with baby, reentering social roles, special occasions, letting go.<br>Turning points were usually associated with stressful situations, and mothers described intrapersonal (e.g. determination) and interpersonal (e.g. social support) coping resources as facilitators of bf maintenance.	20
Schindler-Ruwisch J, et al., 2019 [53] USA (Washington, DC)	Use social cognitive theory and elements of social support as a guiding framework to better understand factors affecting bf initiation and duration.	24 AA women receiving WIC	Semi-structured interviews	Main themes: Influence of others on bf confidence and intention; how bf confers many positive benefits to mother and baby; pervasiveness of bf obstacles; importance of social support.<br>Breastfeeding as best and healthiest. Feeding experiences influenced by barriers at multiple levels: community, interpersonal (social support), and individual constraints (pain, supply, and latching), as well as difficulties of finding resources to overcome challenges. Social support (via emotional, instrumental, appraisal, informational support) from a family member (mother, cousin, sister), friend or partner helped facilitate bf.	19
Schmied V, et al., 2019 [90] Australia	Identify key components of Mother Infant Caring Communities that promote and support bf and early parenting.	35 participants within community conversation workshops including new parents, grandparents, and community members	'Community Conversation' workshop facilitated by appreciative inquiry; Focus group discussion	Themes: PLACE – A community for everyone; A PLACE for children and families; Sometimes a PLACE to bf; The parent room: a hidden and unsafe PLACE to bf<br>BF and parenting are embedded in the places where women and families live. Community spaces including shopping centers, and should be designed to include infants and young children and offer appropriate facilities such as safe and clean parenting rooms.	17
Scott A, et al., 2016 [54] USA (Arkansas)	Understand perceptions, beliefs and experiences about bf in Marshallese migrant mothers.	31 Marshallese migrant mothers living in Arkansas	Focus groups, guided by open-end questions	Themes: Benefits (babies health and quality of milk); Barriers (public shaming and work); Self-efficacy (inadequate milk supply and maternal diet); Activity related affect (child led bf and age to bf); Interpersonal and prior related behavior (familial support in the Marshallese culture); Institutional influences (medical staff and WIC influence).	18
Shloim N, et al, 2015 [77] Israel & UK	Explore how Israeli and UK mothers integrate feeding into their conceptualizations of motherhood, 2-6 months post-partum.	41 women, mostly married or in a committed relationship	Interviews	Themes: A devoted mother who ignores her own needs (always available for my children, children are my entire world); A mother who is available for her infant but acknowledges her needs as well; A struggling mother for whom motherhood is a burden. UK women described more struggles within motherhood whereas a tendency toward idealizing motherhood was observed for Israeli women.	19

Spencer B, et al., 2015 [78] USA	Gain understanding of how AA women experience bf in the context of their day-to-day lives.	AA women; 17 who bf; 6 women who bf and grandmother of bf grandchild	SCQD qualitative methodology; semi-structured interview and focus group interview	Themes: Self-determination; bf is more than best; bf is essential; resisting the naysayers; weathering the storm; resisting stereotypes. Spirituality and bf; what God intended; church and community support. Empowerment; bonding; sense of accomplishment; helping others.<br>Engaging spheres of influence; bf activism; addressing images of the sexual breast vs nurturing breast.	20
Swigart TM, et al., 2017 [55] Mexico	Investigate bf intention, practices, attitudes and beliefs, particularly normative, among low-resource communities in central and southern Mexico.	10 fathers<br>50 mothers<br>44 women leaders<br>321 pregnant women and mothers	Semi-structured interviews (fathers); focus groups (mothers); focus groups (women community leaders)<br>Quantitative survey (pregnant women and mothers)	Bf behavioral beliefs: All participants, including fathers, believed that breast milk was best and healthiest for baby; not useful or nutritious after 6 months. Mothers perceived they were seen as better mothers if they bf when infant was young. Mothers perceived their close community of women (extended family) influenced their infant feeding beliefs and practices. Perceived insufficient milk an accepted reason for ff. Received contradictory and sometimes erroneous info at health centers. Practice of introducing water and teas soon after birth, and small bites of solid food a few months after birth. Social norms appeared to support bf, but not EBF or bf for periods longer than about a year.	18
Tampah-Naah AM, et al., 2019 [85] Ghana	Explore challenges of bf practices by considering spatial, societal and maternal characteristics.	20 mothers aged 15-49 with an infant or child 0-23 months of age	Unstructured in-depth interviews	Key themes were in relation to household chores, work schedules, family influence, low breast milk production, swollen breasts or sore nipples, access to food items and preparation or giving foods.	18
Thepha T, et al., 2018 [81] Thailand	Identify perceived facilitators and barriers to providing EBF for 6 months among bf mothers in northeast Thailand.	30 mothers (aged 20-40 yrs) with children aged 4-6 months, and currently bf or had bf experience	Focus groups (6)	Main themes: bf knowledge, perceptions, maternal circumstances, support, and traditional food.<br>Mother's bf knowledge, intention to bf and social media perceived as facilitators. Perceptions, employment, and formula milk promotion were perceived as barriers. Family, healthcare and traditional food perceived as both facilitators and barriers.	17
Thet MM, et al., 2016 [80] Myanmar	Examine barriers to EBF and how different household members participate in decision-making.	24 mothers with a 6-12 month old and a subset of their husbands (10) and mothers/mothers-in-laws (10)	Semi-structured interviews	Themes: Bf knowledge; bf practices; Reported barriers to EBF; bf support. High levels of knowledge about EBF, but low adherence. Barriers: Family members beliefs that EBF was insufficient; need to return to work; health related problems. Decisions about bf made by the mother herself, with influence of husband support on bf.	19
Thomson G, et al., 2015 [67] UK	Present new insights into how shame-related affects, cognitions, and actions are evident within bf and non-bf women's experiences.	63 women with varied infant feeding experiences	Focus group and semi-structured interviews<br>Using a framework analysis approach and drawing on Lazare's categories of shame	Themes: Exposure of women's bodies and infant feeding methods; undermining and insufficient support; perceptions of inadequate mothering.<br>Findings highlight how bf and non-bf women may experience judgment and condemnation in interactions with hcps as well as within community contexts, leading to feelings of failure, inadequacy and isolation.	18
Thullen M, et al., 2016 [88] USA	Examine co-parenting with regard to infant/toddler feeding practices.	24 sets of co-resident, biological parents with 6 months-3 year olds	Semi-structured interviews	Themes: Mothers were the primary managers of feeding labor. Fathers participated in feeding in different ways and levels starting in infancy and increased involvement in feeding over the first few years requiring an ongoing negotiation around co-parenting related to feeding.	16

Tomori C, et al., 2016 [68]<br>USA & UK	Investigate how the stigmatization of bf shapes bf experiences in society where bf is promoted, but ff remains common.	80 women, most Caucasian	Comparative ethnographic research; 10 in depth interviews with long-term bf women Observation, semi-structured interviews	Findings highlight the divide between perceptions of the ideal of bf and its actual practice and point to the contested moral status of bf in the US and the UK.<br>Some reported lack of support and sympathy from family or hcp for lactation insufficiency and personal feeling of isolation and grief of not bf.	16
Tumilowicz A, et al., 2015 [86]<br>Guatemala	Predict and examine a cumulative sex difference in favor of girls that occurred at approximately 6 months.	24 mothers of mothers from 0-36 months old	Ethnographic interviews included repeated participant observation, field notes, and multiple informal and semi-structured interviews for 18 months	Infant gender differences influenced infant feeding choices. Mothers reported that, compared with female infants, male infants were hungrier, were not as satisfied with breastmilk alone, and required earlier complementary feeding.	17
Wambach K, et al., 2016 [56]<br>USA	Identify perceived benefits and barriers to EBF and levels of acculturation among Mexican American women living in a Midwestern city.	21 Mexican American women living in a midwestern city whom bf for at least 2 weeks	Semi-structured individual interviews (in English or Spanish) using a qualitative descriptive design integrating Pender's Health Promotion Model concepts. The revised Acculturation Rating Scale for Mexican Americans was used to examine acculturation levels	Themes: It is natural that a woman give life and also provide the best food for her baby; bf is ultimately a woman's decision but is influenced by tradition, guidance and encouragement; breast milk is superior but life circumstances can challenge one's ability to bf; social, family (husband/partner if supported bf), and professional support enhanced EBF.<br>Most women EBF, with a few using early supplementation for "insufficient milk production". Findings support the need for culturally competent and individualized lactation care.	18
Wandel M, et al., 2016 [57]<br>Norway	Explore infant feeding practices among Somali-born mothers in Norway, and the ways in which they navigate among different information sources.	Mothers of children 6, 12 and 24 months of age (21 in interviews and 22 in focus groups)	In-depth interviews and focus groups	Mothers had positive attitude toward bf, but were unfamiliar with the concept of EBF. Early intro of water and infant formula was a common practice that interfered with EBF. Mothers experienced challenges of dealing with conflicting recommendations (conflict between family member and hcp advice, leaving mothers to feed insecure about how to feed); experiences with hcp's varied as did expectations regarding infant feeding. Mothers navigated among different sources of info, considering traditional values, experiences, habits, and research-based knowledge.	17
Wanjohi M, et al., 2017 [60]<br>Africa (Kenya)	Describe cultural and social beliefs and practices that influence bf in two urban slums.	<u>FGD:</u> 2 village elders, 3 young mothers, 3 older mothers and 2 community health workers<br><u>KII</u>: 3 women group leaders, 2 youth group leaders, 2	10 focus group discussions, 11 key informant interviews and 19 in-depth interviews	Results aligned with WHO recco's: colostrum is medicine; EBF is beneficial, but there are exceptions. Association of breast milk with intellectual development and good child health.<br>Results not aligned with WHO recco's: Colostrum considered as 'dirty' or 'taboo', a curse 'bad omen' in some situations, a fear of the 'evil eye' (malevolent glare which is believed to be a curse associated with witchcraft) when bf in public, and bf being associated with sagging breasts. Long duration of bf is counter-productive.	17

		traditional birth attendants, 2 religious leader and 2 healthcare workers<br>IDI: 3 pregnant women, 9 bf mothers, 3 non-bf mothers and 4 HIV positive mothers			
Wennberg AL, et al., 2017 [58]<br>Sweden	Analyze perceptions of mothers, who are discussing the topic on web forums, about introducing infant formula.	2 online parenting web forums where women posted their views on introducing infant formula	Qualitative, descriptive, cross-sectional study using online data. Analyzed using qualitative content analysis	Main themes: balancing between social expectations and confidence in your parental ability. Sub themes: striving to be a good mother (bf is best, at any cost); promotion of bonding with infant. Striving for your own well-being; subthemes included pain and problems, feeling tired and losing feeding, and getting into control to feel secure. Striving to discover your own path; subthemes included negotiating pros and cons, dealing with pressure, impact of others, and standing up for your decision.	17
Yate ZM, 2017 [71]<br>UK	Describe Bf aversion and agitation of women and their experiences with bf.	434 women, internationally	Survey questionnaire using thematic and inductive content analysis, with qualitative interpretive description	Findings shed lights on an experienced phenomenon of aversion and agitation while bf, which varies in form, severity and duration. A number of mothers who experience aversion still continue to bf, but had feelings of guilt and shame while also experiencing confusion around those feelings.	16
Yue A, et al., 2016 [98]<br>China	Elucidate some of the fundamental causes of poor complementary feeding practices that may lead to anemia among children in rural China.	60 primary caregivers	Semi-structured interviews	Poor complementary feeding practices are common, and impeded by 1) absence of understanding topics related to infant health and nutrition under caregivers, 2) inadequate sources of info on these topics. Inconsistent info, leaving caregivers confused about credibility of sources.	17
Zhang X and Benton L, 2019 [101]<br>UK (England)	Explore association between acculturation and feeding practices among new Chinese immigrant mothers living in England.	Chinese immigrant mothers in England<br>N=32 (questionnaire)<br>N=15 (interviews)	Questionnaire<br>Semi-structured interviews	Chinese immigrant mothers balanced western and Chinese feeding practices to combat feeding and culture conflict. Those high in integration were more likely to respond to satiety and attention; those described as marginalized were more likely to indulge their children. Mothers that were more culturally separated were more likely to restrict the food quality offered to their children.	18
Zhang K, et al., 2015 [87]<br>China	Elicit and compare mothers' and hospital staff perceptions of the reasons that shaped decision to ff.	50 mothers<br>33 hospital staff	In-depth interviews (mothers) and focus group discussions (hospital staff)	Themes: insufficient breast milk; influence of maternal mothers or mothers-in-law, or 'confinement ladies'; inadequate bf facilities; limited flexibility at work.	16

†CASP (Critical Appraisal Skill Programme for qualitative research) score based on maximum of 20 points; see associated text.
Abbreviations: AA=African American, bf=breastfeed, EBF=exclusive breastfeed, ff=formula feed, hcp=health care provider, intro=introduction, recco's=recommendations
